# Management of Complex Dorsal Dislocation of the Index Finger Metacarpophalangeal Joint by a Combined Open Dorsal and Volar Approach: A Case Report

**DOI:** 10.7759/cureus.91271

**Published:** 2025-08-30

**Authors:** Hongzhi Zhu, Jingwen Ng, Mala Satku

**Affiliations:** 1 Orthopaedic Surgery, Woodlands Health, Singapore, SGP; 2 Hand and Reconstructive Microsurgery, Tan Tock Seng Hospital, Singapore, SGP

**Keywords:** early open reduction, index finger, kaplan lesion, metacarpophalangeal joint, open surgical approach

## Abstract

Dorsal dislocations of the metacarpophalangeal joint (MCPJ) are a rare occurrence, often caused by falling on the outstretched hand with hyperextension of the MCPJ. MCPJ dislocations can be complex due to the interposition of soft tissue, making closed reduction not possible.

This report describes a case of complex dorsal dislocation of the index finger MCPJ managed by a combined open dorsal and volar approach with a good functional outcome. The patient was followed up at two weeks and three months postoperatively. He reported no pain, minimal stiffness, and a stable index finger MCPJ at three months postoperatively.

Early recognition of complex MCPJ dislocation is crucial, and repeated closed reduction attempts should be avoided. Early surgery in such cases enables the patient to achieve a stable MCPJ with good functional outcomes.

## Introduction

Dislocations of the metacarpophalangeal joint (MCPJ) are uncommon and are often caused by falling on the outstretched hand with hyperextension of the MCPJ [1,2], resulting in a dorsal dislocation. The complex index finger MCPJ dislocation was described by Kaplan in 1957, where the metacarpal head buttonholes volarly, with the volar plate interposed between the base of the proximal phalanx and the metacarpal head [3]. Kaplan’s lesion is a rare occurrence, but it is crucial for early recognition, as closed reduction will not be possible in such cases.

This report describes a case of complex dorsal dislocation of the index finger MCPJ and aims to review the literature available on techniques for the reduction of MCPJ dislocations and surgical approaches for irreducible dislocations.

## Case presentation

**Figure 1:**
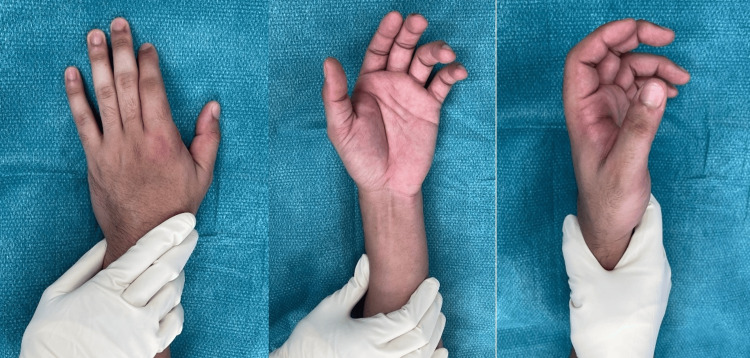
Clinical photos of the patient’s right hand pre-reduction with the classical bayonet position of the index finger and dimpling of the volar skin

A 24-year-old male was seen in the Accident & Emergency (A&E) department after a fall from his bicycle onto his outstretched right hand. He had pain in his right index finger and noticed a deformity in his index finger. He did not sustain any other injuries. On examination in the A&E, the patient had tenderness and swelling over his right index finger metacarpophalangeal joint (MCPJ), associated with an extension deformity of the proximal phalanx (Figure 1).

**Figure 2:**
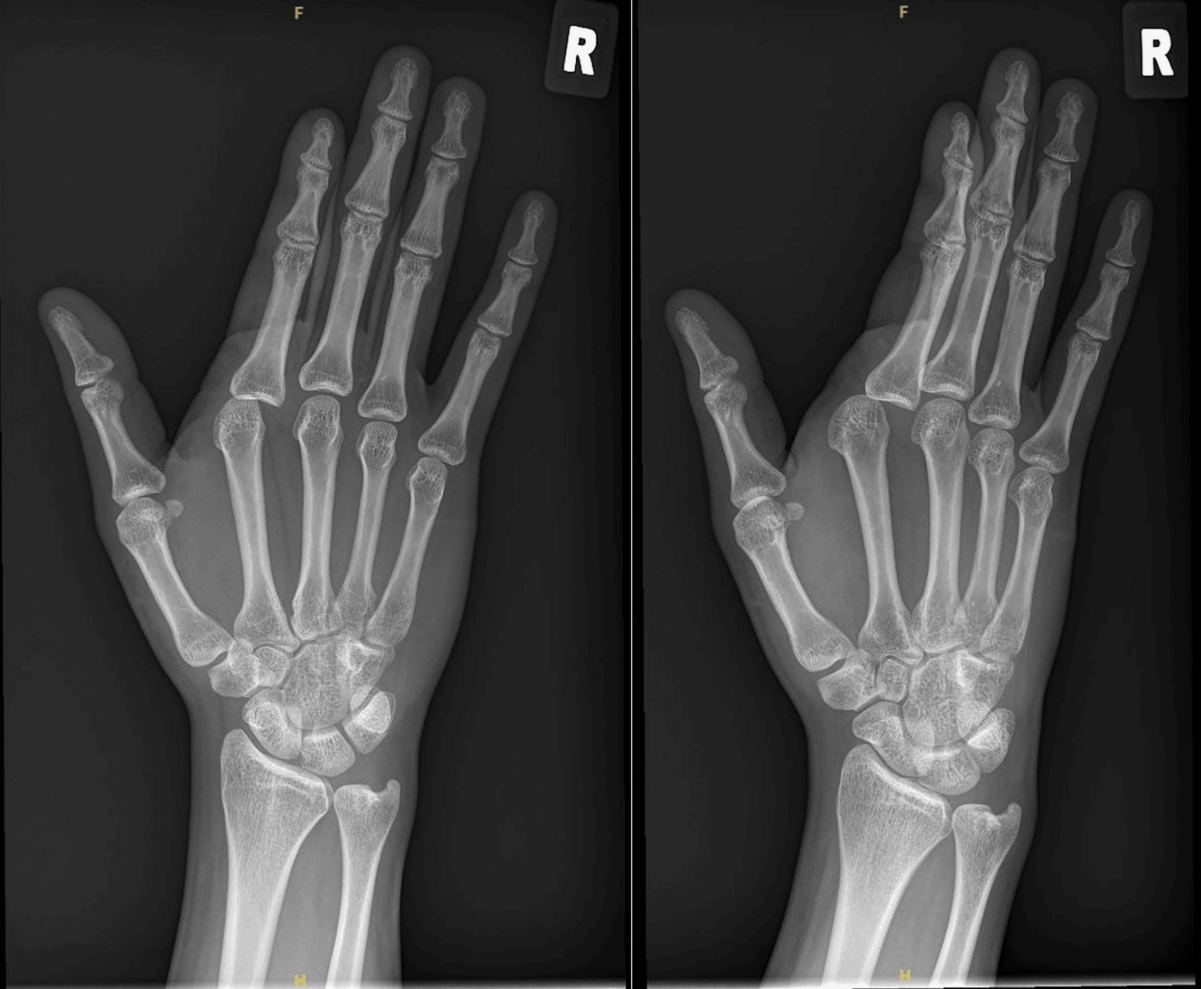
Plain radiographs of the patient’s right hand showing right index finger MCPJ dislocation MCPJ: metacarpophalangeal joint

There were no open wounds, and the index finger sensation and vascularity were normal. Plain radiographs showed a dorsal-ulnar dislocation of the index finger MCPJ; no fractures were noted (Figure 2).

Closed manipulation and reduction under sedation were attempted twice and were unsuccessful. The case was escalated to the Hand Surgery specialist on duty. During immediate onsite specialist review of the patient, a complex dorsal dislocation of the right index finger MCPJ was recognized. The patient was counselled and listed for open reduction in the emergency operating theatre.

Surgical approach

**Figure 3:**
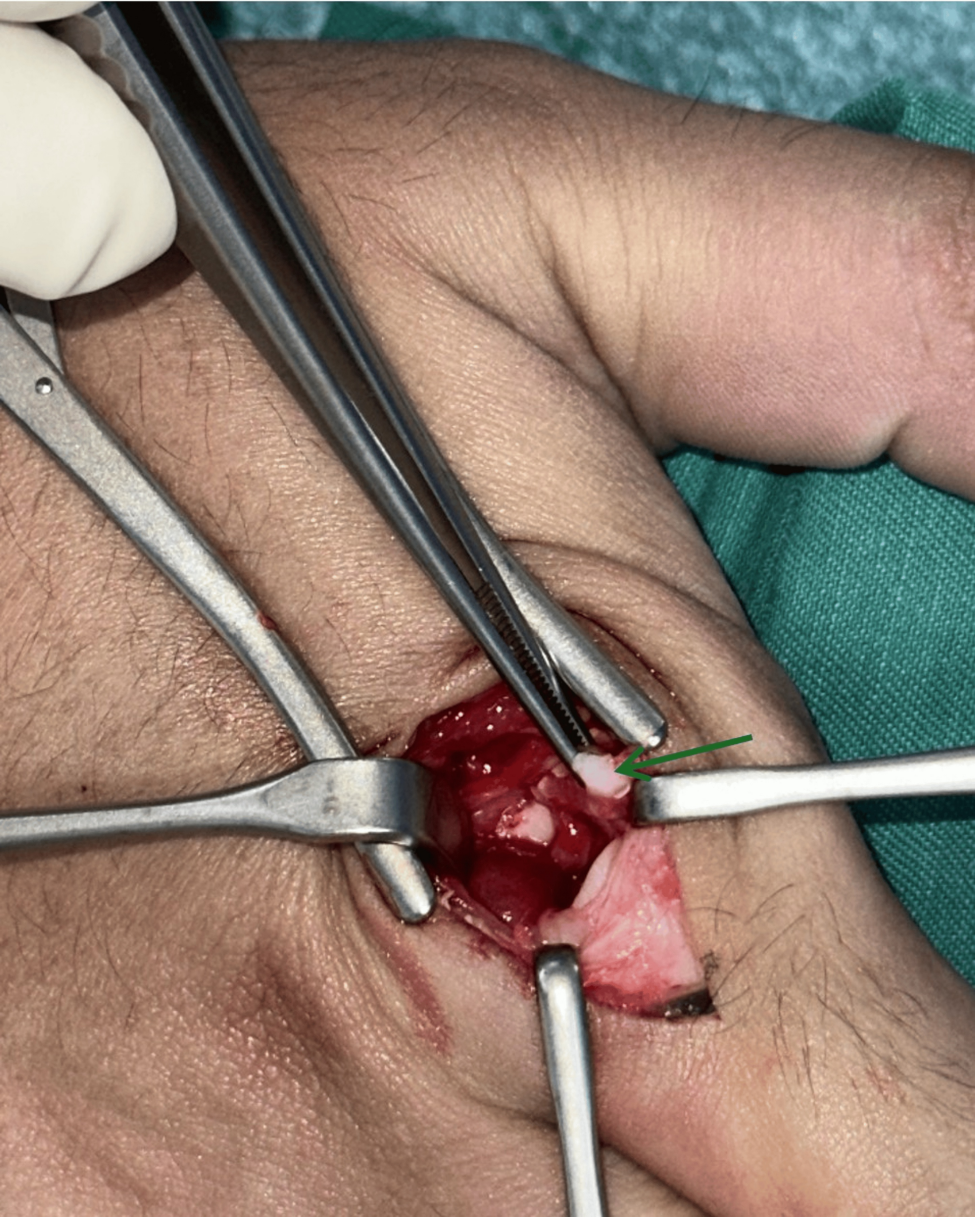
Dorsal approach to the index finger MCPJ, showing an interposed volar plate within the MCPJ MCPJ: metacarpophalangeal joint

The surgery was conducted under general anesthesia in the supine position. An upper arm tourniquet was applied, and the right hand was cleaned and draped via routine aseptic technique. A longitudinal dorsal incision was made centered over the index finger MCPJ. Extensor tendons were retracted ulnarly. The MCPJ capsule was divided longitudinally (Figure 3).

**Figure 4:**
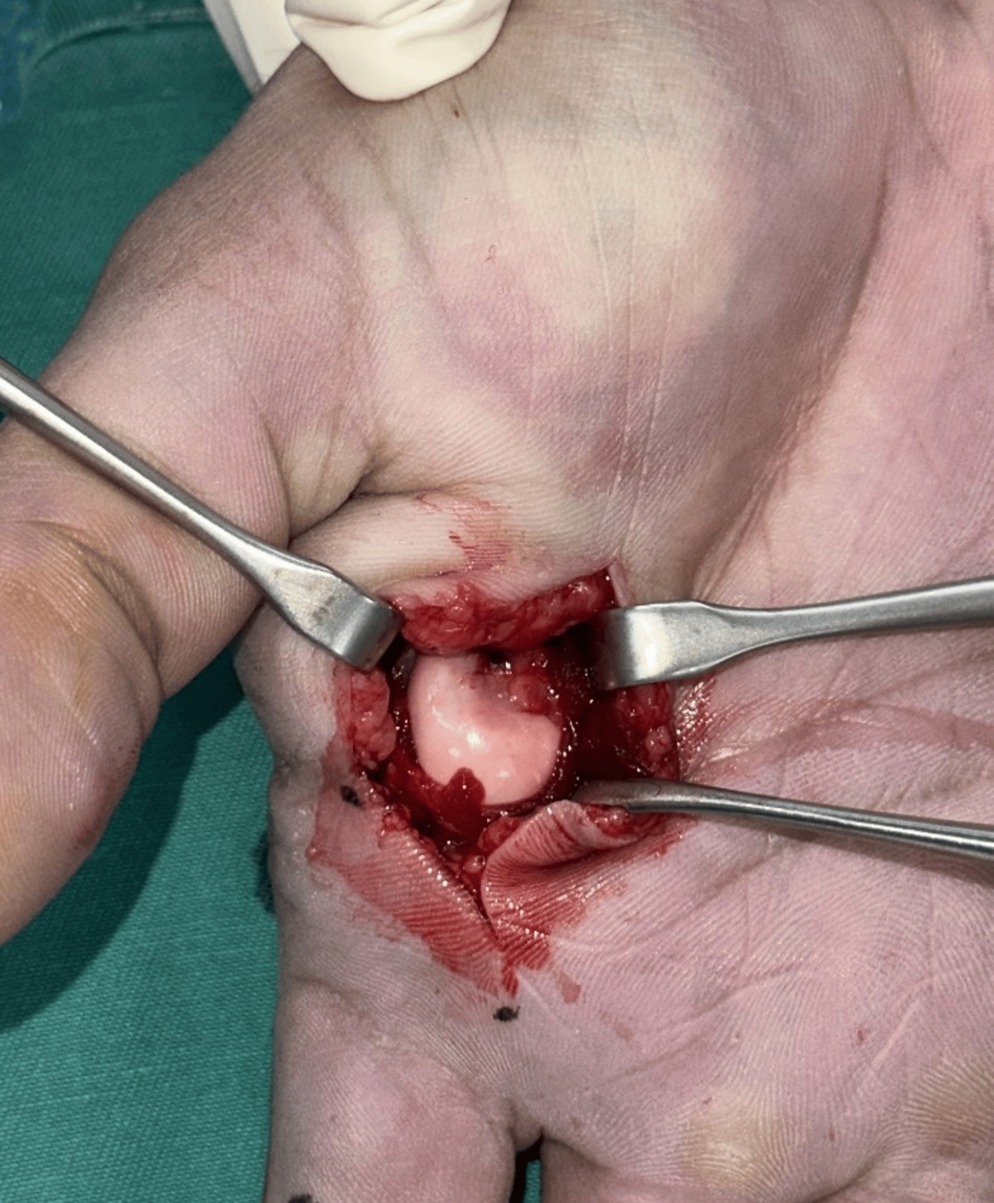
Volar approach to the index finger MCPJ, showing the buttonholed second metacarpal head MCPJ: metacarpophalangeal joint

The interposed volar plate could not be delivered using the freer elevator, and reduction was still not possible. A volar Bruner’s incision centered over the index finger MCPJ was made (Figure 4). 

The metacarpal head was found to be volar to the transverse metacarpal ligament, the latter creating a noose with the flexor tendon ulnarly and the lumbrical tendon radially.

**Figure 5:**
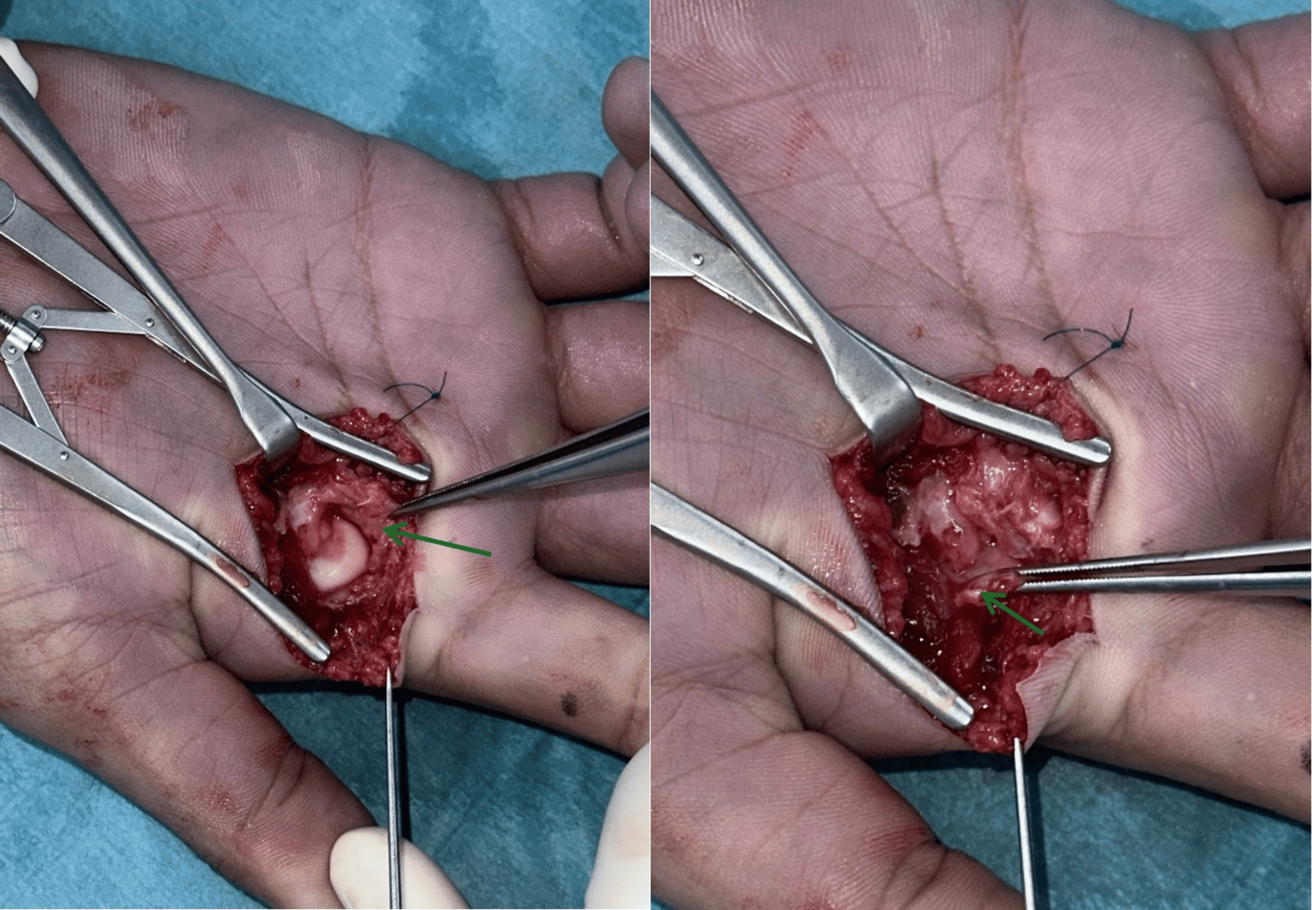
Intra-operative clinical photo showing division of the transverse metacarpal ligament, as identified by the forceps

**Figure 6:**
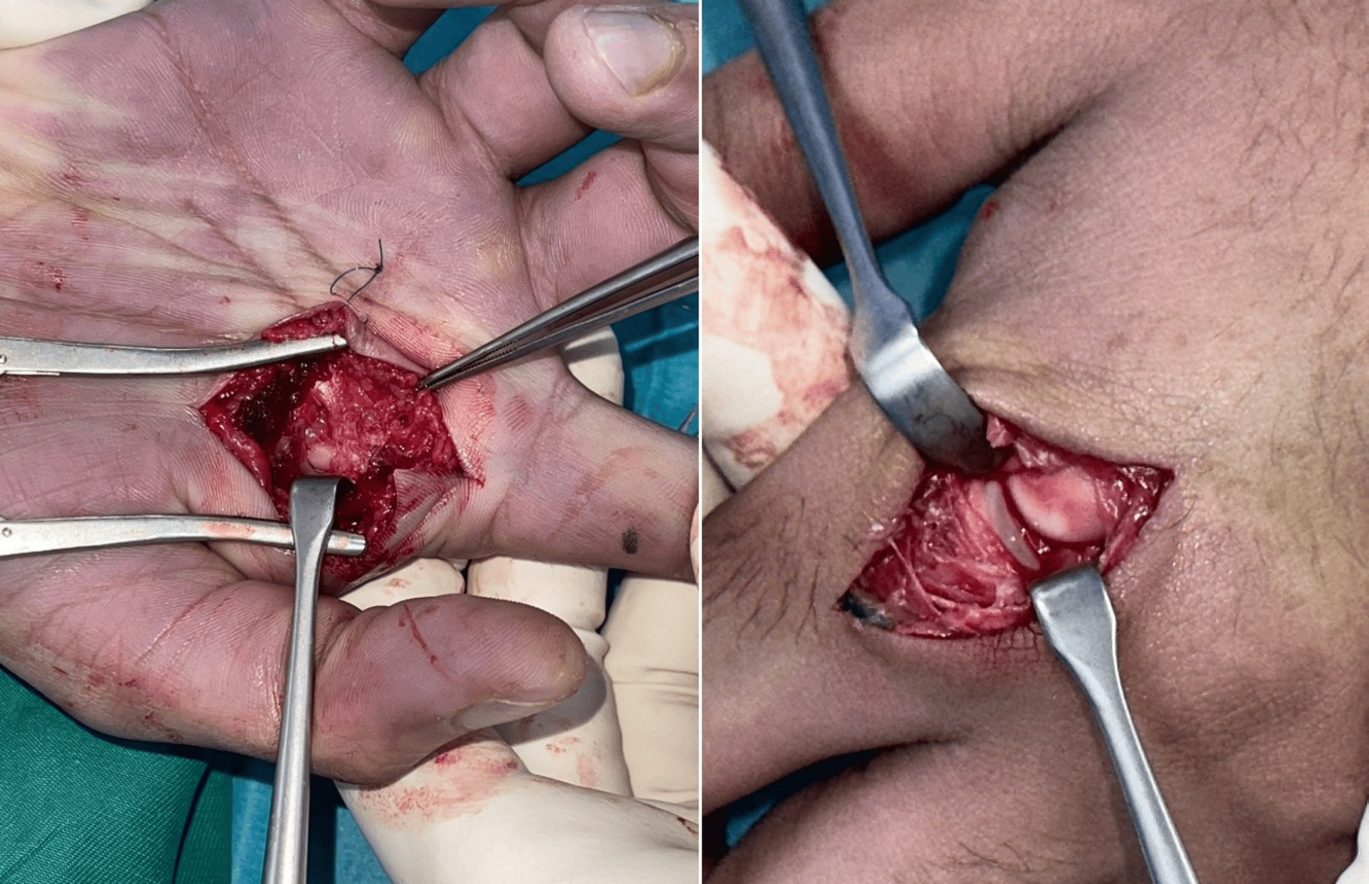
Intra-operative clinical photo showing the reduced MCPJ and repaired transverse metacarpal ligament MCPJ: metacarpophalangeal joint

The transverse metacarpal ligament was divided (Figure 5), and the MCPJ was reduced (Figure 6).

**Figure 7:**
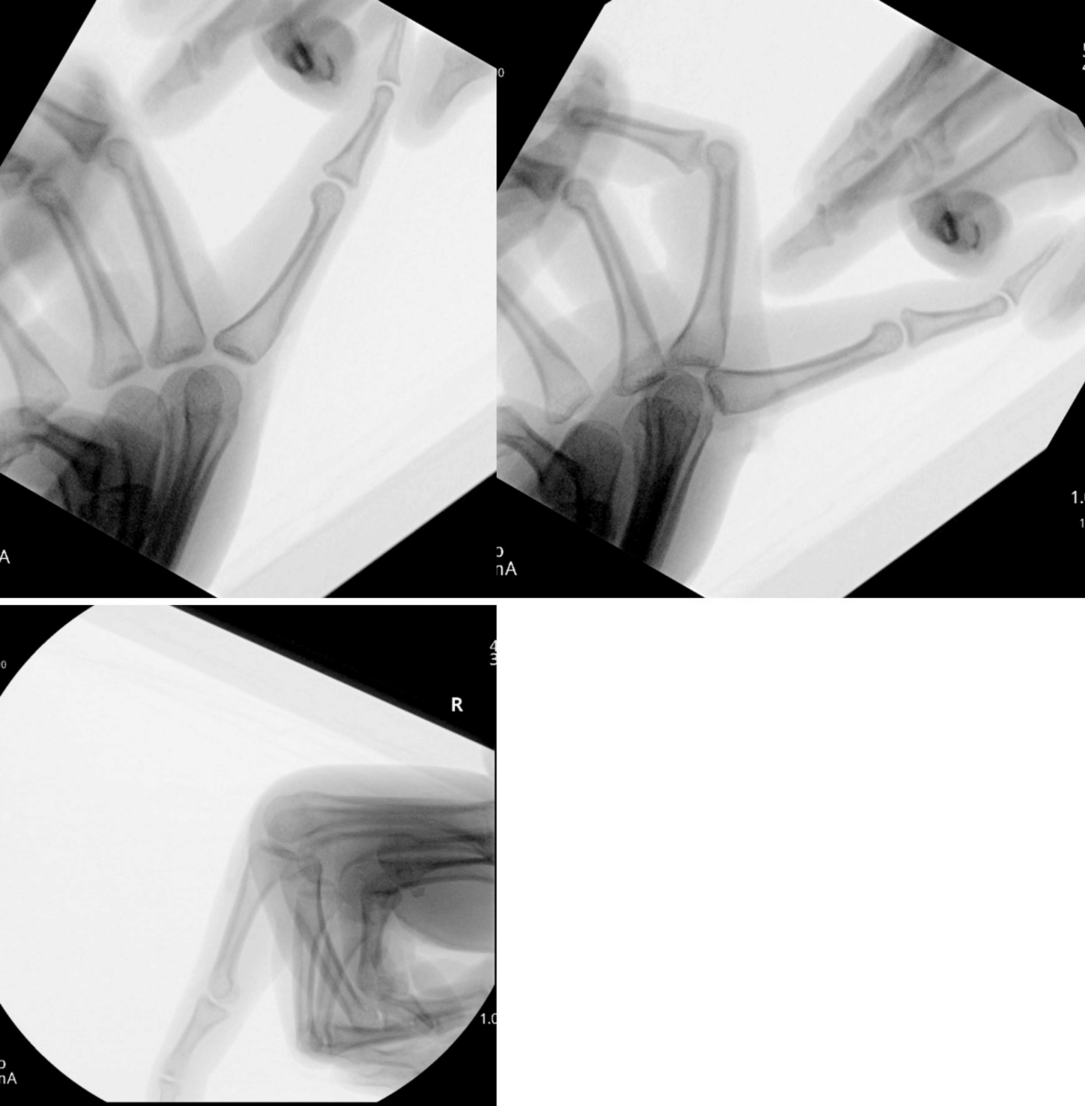
Intra-operative image intensifier images showing the enlocated index finger MCPJ stable in extension and flexion MCPJ: metacarpophalangeal joint

The reduction and joint stability were confirmed clinically and radiographically (Figure 7).

The transverse metacarpal ligament and dorsal capsulotomy were repaired, and the wounds were closed in layers. An intrinsic plus dorsal backslab was placed postoperatively.

Follow-up

The patient’s wounds were well-healed for two weeks postoperatively, and the stitches were removed. The patient was placed on a removal intrinsic-plus splint for four weeks and commenced on rehabilitation with the hand occupational therapist. He reported no pain, minimal stiffness, and a stable index finger MCPJ at three months postoperatively.

## Discussion

The MCPJ is a condyloid joint, which allows flexion, extension, abduction, adduction, and circumduction. The MCPJ is stabilized by the proper and accessory collateral ligaments, volar plate, and dorsal capsule. Surrounding soft tissue, like the transverse metacarpal ligament, the flexor tendons of the index finger, and the lumbrical muscle, may entrap the metacarpal head in a complex MCPJ dislocation [4].

Index finger MCPJ dislocation, as described by Kaplan, is a rare occurrence. The dislocation tends to occur dorsally, as the joint capsule is weakest and thinnest dorsally [2,3]. On physical examination, one may notice dimpling of the skin in the proximal palmar crease volarly, and there is bayonet positioning of the proximal phalanx dorsal to the metacarpal shaft. Diagnosis can be confirmed with plain radiographs of the hand and affected finger, including a true lateral view of the joint.

Potential complications may arise from MCPJ dislocations such as joint stiffness, persistent pain, post-traumatic osteoarthritis, or osteonecrosis. These complications are associated with repeated failed attempts at closed reduction, prolonged dislocation, and traumatic open reduction [5].

**Figure 8:**
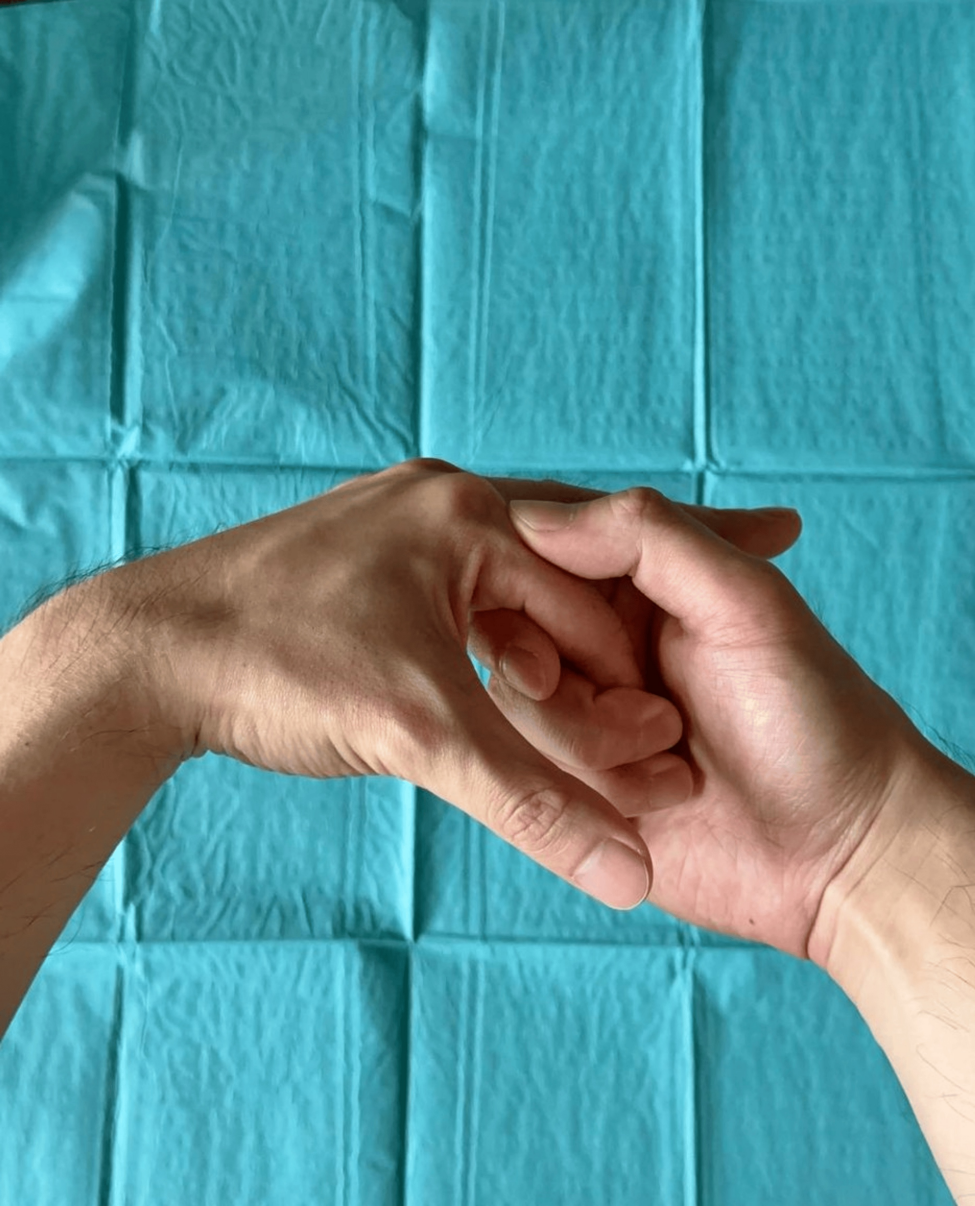
Reduction of a dorsal IF MCPJ dislocation with the wrist in slight flexion Pressure is applied at the base of the proximal phalanx to slide it over the metacarpophalangeal joint (MCPJ). Technique: [6]

MCPJ dislocation is considered simple when there is no interposition of the volar plate or other soft tissue, allowing for closed reduction. In such cases, the MCPJ is usually subluxed, rather than completely dislocated, with the base of the proximal phalanx still in partial contact with the metacarpal head. Closed reduction is performed by applying direct pressure over the dorsal aspect of the proximal phalanx, with the wrist in flexion to relieve tension of the intrinsic and extrinsic flexors (Figure 8) [2,6].

The MCPJ dislocation is described as complex when there is interposition of the volar plate between the proximal phalanx and the metacarpal head, making closed reduction not possible [3]. Certain pitfalls during closed reduction may convert a simple dislocation to a complex one. Application of longitudinal traction or repeated failed closed attempts during closed reduction may pull the volar plate into the MCPJ, converting it into an irreducible complex MCPJ and may result in complications such as post-traumatic arthritis or osteonecrosis [5,7]. Early recognition of complex MCPJ dislocations is important and should be considered a surgical emergency [8,9]. We recommend that closed reduction not be attempted more than once, particularly when the patient is first seen in the Emergency Department, and if there is suspicion of complex MCPJ dislocation or if closed reduction has failed, to obtain an urgent specialist consult.

There are two commonly described surgical approaches to open reduction for MCPJ dislocation. The dorsal approach utilizes a midline incision over the MCPJ and aims to push the entrapped volar plate out using the freer elevator [10]. The advantage of this approach is that there is a lower risk of injury to the neurovascular bundle [10]. However, there may be a need to split the volar plate to facilitate reduction, and this may delay recovery [11]. On the other hand, the volar approach provides better access to the volar plate and surrounding ligaments and tendons, which may require release in a complex MCPJ dislocation [2,3,12]. However, there is a higher risk of injury to the neurovascular bundle [12].

We recommend the dorsal approach as the first-line option in dorsal index finger MCPJ dislocation, as it is a safe and relatively simple approach that avoids risks of damaging the neurovascular bundle. On the other hand, the volar approach offers better visualization and access to the MCPJ, enabling the surgeon to perform soft tissue releases in cases where the MCPJ is irreducible dorsally. Our case illustrates the decision-making algorithm for our surgical approach, where the index finger MCPJ was irreducible dorsally, and a combined volar approach was necessary to allow for soft tissue release and safe reduction of the MCPJ. This case contributes to existing literature by describing a useful algorithm in the surgical approaches for the open reduction of dorsal index finger MCPJ dislocation, with satisfactory clinical outcomes.

## Conclusions

It is crucial to recognize a complex dislocation and avoid repeated closed reduction attempts. Open reduction is indicated in complex MCPJ dislocation, and the dorsal approach for dorsal dislocation is typically safe and simple to perform. However, an additional volar approach is needed in cases where the dorsal approach alone is inadequate in achieving safe and stable reduction of the MCPJ. Early recognition with early surgery in complex index finger MCPJ dislocation enables the patient to achieve a stable MCPJ with good functional outcomes. This case report contributes to existing literature by emphasizing the importance of early recognition, early surgery, and illustrates the decision-making algorithm in surgical approaches in complex index finger MCPJ dislocation. Further large-scale studies on the outcomes of open reduction for index finger MCPJ dislocation should be conducted.
